# Genome instability-derived genes as a novel prognostic signature for lung adenocarcinoma

**DOI:** 10.3389/fcell.2023.1224069

**Published:** 2023-08-16

**Authors:** Xu Zhang, Tak-Wah Lam, Hing-Fung Ting

**Affiliations:** Department of Computer Science, The University of Hong Kong, Pokfulam, Hong Kong SAR, China

**Keywords:** genome instability, lung adenocarcinoma, somatic mutations, copy number variation, fusion genes, overall survival prediction

## Abstract

**Background:** An increasing number of patients are being diagnosed with lung adenocarcinoma, but there remains limited progress in enhancing prognostic outcomes and improving survival rates for these patients. Genome instability is considered a contributing factor, as it enables other hallmarks of cancer to acquire functional capabilities, thus allowing cancer cells to survive, proliferate, and disseminate. Despite the importance of genome instability in cancer development, few studies have explored the prognostic signature associated with genome instability for lung adenocarcinoma.

**Methods:** In the study, we randomly divided 397 lung adenocarcinoma patients from The Cancer Genome Atlas database into a training group (*n* = 199) and a testing group (*n* = 198). By calculating the cumulative counts of genomic alterations for each patient in the training group, we distinguished the top 25% and bottom 25% of patients. We then compared their gene expressions to identify genome instability-related genes. Next, we used univariate and multivariate Cox regression analyses to identify the prognostic signature. We also performed the Kaplan–Meier survival analysis and the log-rank test to evaluate the performance of the identified prognostic signature. The performance of the signature was further validated in the testing group, in The Cancer Genome Atlas dataset, and in external datasets. We also conducted a time-dependent receiver operating characteristic analysis to compare our signature with established prognostic signatures to demonstrate its potential clinical value.

**Results:** We identified GULPsig, which includes *IGF2BP1*, *IGF2BP3*, *SMC1B*, *CLDN6*, and *LY6K*, as a prognostic signature for lung adenocarcinoma patients from 42 genome instability-related genes. Based on the risk score of the risk model with GULPsig, we successfully stratified the patients into high- and low-risk groups according to the results of the Kaplan–Meier survival analysis and the log-rank test. We further validated the performance of GULPsig as an independent prognostic signature and observed that it outperformed established prognostic signatures.

**Conclusion:** We provided new insights to explore the clinical application of genome instability and identified GULPsig as a potential prognostic signature for lung adenocarcinoma patients.

## 1 Introduction

Lung cancer is one of the most frequently diagnosed cancer types and the leading cause of cancer-related deaths worldwide (Bray et al., 2018). Lung adenocarcinoma, the most prevalent subtype of lung cancer, accounts for 40% of all lung cancer types and continues to increase (Nagy-Mignotte et al., 2011; Myers and Wallen, 2023). However, there has been limited progress in improving prognostic outcomes and enhancing survival rates for lung adenocarcinoma patients (Travis et al., 2015). Therefore, a prognostic signature for lung adenocarcinoma would be highly valuable for clinical management.

Genome instability, one of the hallmarks of cancer, is regarded as a contributing factor for other hallmarks to acquire functional capabilities that allow cancer cells to survive, proliferate, and disseminate (Hanahan and Weinberg, 2011). Moreover, genome instability has been proved to be a vital prognostic factor associated with cancer progression and survival (Suzuki et al., 2003; Negrini et al., 2010). Signatures related to genome instability have been identified in certain types of cancers. For example, a prognostic signature including 11 genome instability-derived genes was identified for triple-negative breast cancer (Guo and Wang, 2021). A signature composed of five prognostic lncRNAs associated with genome instability was identified in endometrial cancer (Liu et al., 2022). Woo et al. (2021) identified circulating tumor DNA–genomic instability I-scores as a prognostic marker for pancreatic cancer survival, and Sun et al. (2021) found a prognostic signature composed of six lncRNAs associated with genome instability for gastric cancer. However, few studies have explored the association between genome instability and survival prediction in lung adenocarcinoma.

Genome instability is defined as an increased propensity for genomic alterations, including somatic mutations, copy number variations, and fusion genes (Shen, 2011). Patients with various genomic alterations may be affected differently. We assumed that there could be a signal associated with genome instability that plays a role in tumor development and drug resistance and that this potential signal could serve as a prognostic signature to predict the overall survival of lung adenocarcinoma patients. To identify the signature, we proposed comparing the gene expression of patients with various genomic alternations, which can be measured by integrating somatic mutations, copy number variations, and fusion genes.

In the study, we quantified genomic alternations by calculating the cumulative counts of somatic mutations, copy number variations, and fusion genes. We then selected the top 25% of patients as the genomic stable-like (GS) group and the bottom 25% as the genomic unstable-like (GU) group from the ranked cases in ascending order. By comparing the gene expressions between the GS and GU groups, we identified 42 genes associated with genome instability. Furthermore, from these 42 genes, we identified GULPsig, composed of *IGF2BP1*, *IGF2BP3*, *SMC1B*, *CLDN6*, and *LY6K*, as a prognostic signature for predicting overall survival. Subsequently, we constructed a risk model with GULPsig and divided the patients into high- and low-risk groups based on the score of the risk model to predict the overall survival of lung adenocarcinoma patients. We further evaluated the independence of GULPsig as a prognostic signature and compared its performance with established prognostic signatures. Overall, our study found GULPsig as an independent prognostic signature for overall survival prediction in clinical management for lung adenocarcinoma patients.

## 2 Materials and methods

### 2.1 Data acquisition

The genomic alterations, gene expression profiles, and corresponding clinical data of lung adenocarcinoma from The Cancer Genome Atlas (TCGA) Pan-Cancer Atlas dataset were obtained from the cBioPortal database (Cerami et al., 2012; Gao et al., 2013). The dataset includes somatic mutations and fusion genes of 566 patients, copy number variations of 511 patients, mRNA expressions of 510 patients, and clinical data of 566 patients. After data integration, data of 397 patients were used to construct and evaluate the prognostic risk model. The gene expression data were batch-normalized from Illumina HiSeq_RNASeq V2. In addition, we collected the expression data and corresponding clinical data of 133 patients in GSE42127 (Hight et al., 2020) and 85 patients in GSE30219 (Rousseaux et al., 2013) from the Gene Expression Omnibus (GEO) database to validate the performance of the risk model. To access the GEO data, we used the R package “GEOquery” (Davis and Meltzer, 2007).

### 2.2 Identification of genome instability-related genes

To visualize the frequency of somatic mutations, copy number variations, and fusion genes for each gene in each patient, we developed a binary matrix with genes in rows and patients in columns to represent the genome events independently for the 397 patients in the TCGA dataset. Then, we computed the sum of the three binary matrices to obtain the total genome events for each patient. Based on the results, we sorted the patients and classified the top 25% as the genomic stable-like (GS) group and the bottom 25% as the genomic unstable-like (GU) group.

For the gene expression data, we used a log2 (x + 1)-transformed RSEM normalized count and then performed quantile normalization on the data. Only genes that had 10 counts or more in at least 10 samples were kept for differential expression analysis. The R package “limma” (Ritchie et al., 2015) was used to compare the gene expression between the GU and GS groups. The differentially expressed genes (DEGs) were identified with a Benjamini–Hochberg (BH)-adjusted *p*-value <0.05 and logFC (fold-change) > 2.

### 2.3 Functional enrichment analysis of genome instability-related genes

The functions enrichGO and enrichKEGG in the R package “clusterProfiler” (Wu et al., 2021) were applied for enrichment analysis and annotation of the Gene Ontology (GO) and Kyoto Encyclopedia of Genes and Genome (KEGG) pathways for DEGs. We identified the biological properties of the DEGs and visualized the DEG enrichment of molecular function (MF), cellular component (CC), biological process (BP), and KEGG pathways. A BH-adjusted *p*-value <0.05 was considered statistically significant.

### 2.4 Construction and validation of the risk model

The 397 patients in the TCGA dataset were randomly divided into a training group and a testing group, with a balanced proportion of living and deceased overall survival status. We used the 199 patients in the training group to identify the prognostic signature and construct the risk model. The univariate Cox proportional hazards regression analysis was applied to evaluate the relationship between the expression level of each gene and the overall survival of patients. Only a gene with a *p*-value of <0.05 was regarded as a statistically significant survival predictor. Next, we applied the multivariate Cox regression model to construct the risk model. To evaluate the risk of each patient, we used the following risk score formula:

$$Overall\;risk\;score\;\left(ORS\right)=\sum_{i=1}^{N}\left({Coef}_{i}\times {Expr}_{i}\right),$$

where N is the total number of genes, Expr is the normalized gene expression value, and Coef is the estimated regression coefficient value of the gene. We calculated the ORS for each patient with this formula and then sorted them. To divide the patients into high- and low-risk groups based on ORS, we used the function “surv_cutpoint” in the R package “survminer” to determine the optimal cut-off threshold. The Kaplan–Meier (KM) survival analysis and a log-rank test were performed with the R package “survival” and “survminer.” Next, we used the R package “e1071” to train a support vector machine (SVM) classifier using the expression data of selected genes and the identified groups. The classifier’s performance was evaluated with 10-fold cross-validation using the R package “caret.” Similarly, the aforementioned operations were conducted on the 198 patients in the testing group and 397 patients in the TCGA dataset. To further validate the performance of the risk model, we applied the same operations to 133 patients in GSE42127 (Tang et al., 2013; Hight et al., 2020) and 85 patients in GSE30219 (Rousseaux et al., 2013). Additionally, we performed a univariate and multivariate Cox proportional hazards regression, as well as data stratification analysis, to identify the independence of ORS as an independent prognostic indicator.

### 2.5 Evaluation of the risk model

We applied the TCGA dataset to construct the established benchmarks following the instructions in the respective publications. The R package “survivalROC” was used to conduct a time-dependent receiver operating characteristic (ROC) analysis and calculate the area under the curve (AUC). The performance of the risk model was assessed by measuring the AUC.

### 2.6 Statistical analysis

A Wilcoxon rank-sum test was performed on the continuous variables, and a chi-square test was performed for categorical data to compare the differences, as appropriate. Statistical significance was defined when *p* < 0.05. Statistical analysis and visualization were performed with R version 4.2.1.

## 3 Results

### 3.1 Identification of genome instability-related genes

**FIGURE 1:**
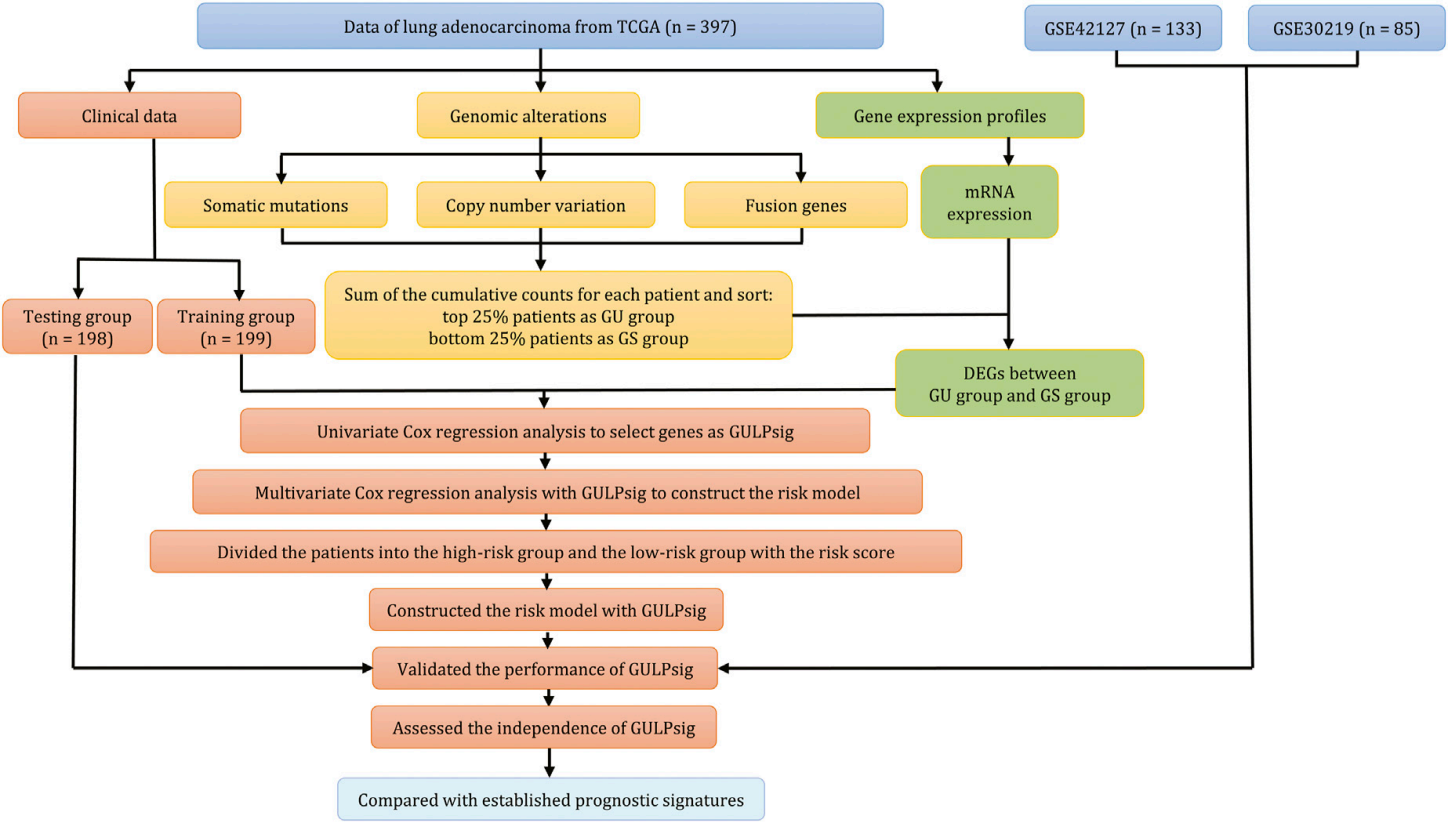
Methodology of the study.

**FIGURE 2:**
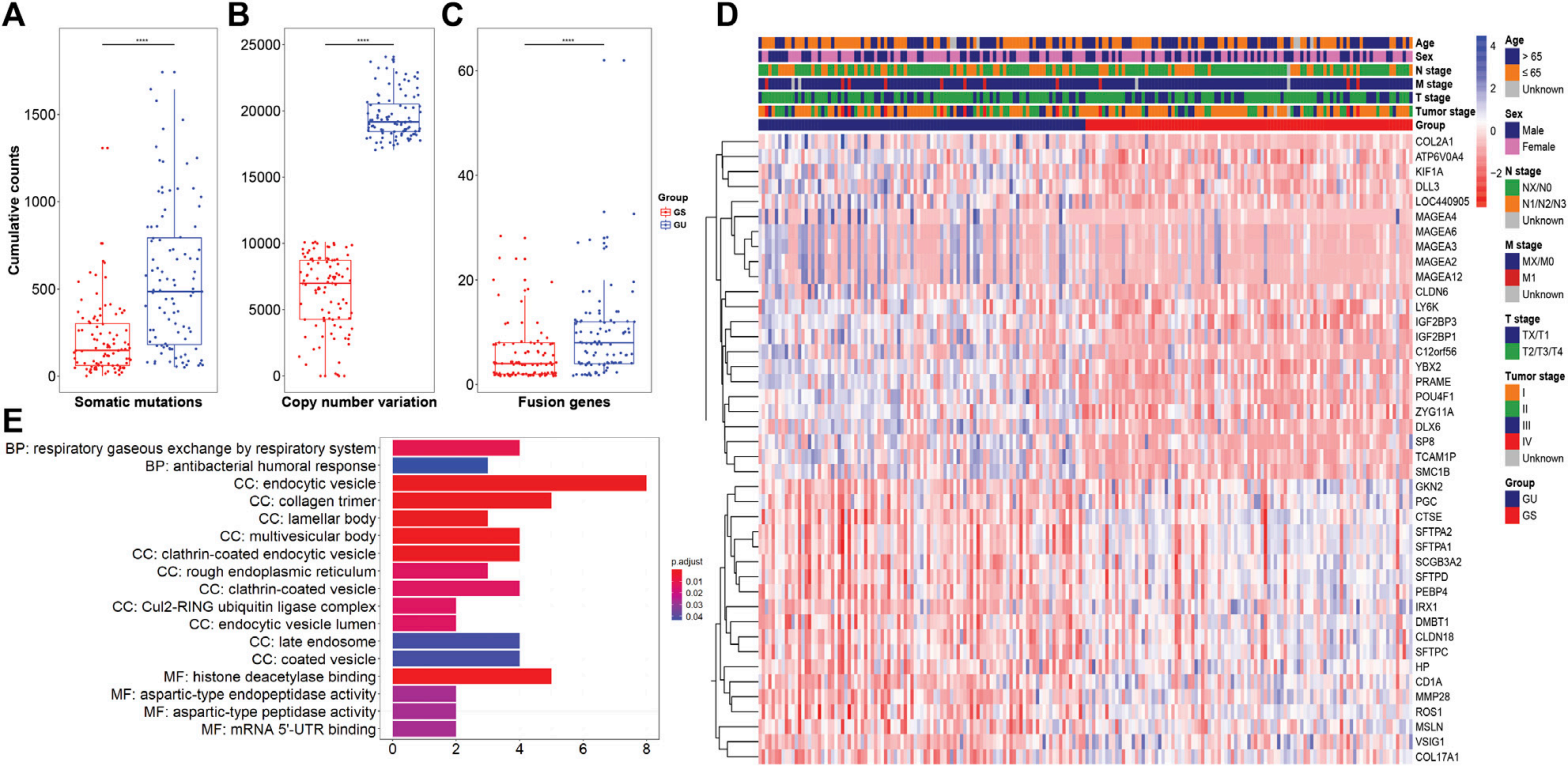
Identification of genome instability-related genes in lung adenocarcinoma. **(A–C)** Boxplots of cumulative counts for somatic mutations, copy number variations, and fusion genes between the GS and GU groups. **(D)** Hierarchical cluster of the patients in the GS and GU groups with the expression of 42 genome instability-related genes. **(E)** GO terms for functional enrichment of the 42 genome instability-related genes.

Figure 1 illustrates the methodology of the study. We involved 397 patients in the TCGA dataset and briefly addressed the clinical characteristics in Table 1. Furthermore, we calculated the cumulative counts of genomic alterations for each patient, consisting of somatic mutations (Figure 2A), copy number variations (Figure 2B), and fusion genes (Figure 2C). We also summarized the genomic alterations and sorted the counts in ascending order. The top 25% of the lung adenocarcinoma patients (n = 99) were defined as the GS group, and the bottom 25% (n = 99) were defined as the GU group. We performed a statistical analysis of the clinical characteristics of the GU and GS groups and found no association (Supplementary Table S1, *p* > 0.05).

**TABLE 1:** Clinical information for patients with lung adenocarcinoma.

Categories		Training group	Testing group	TCGA dataset	*p*-value *
		(n = 199)	(n = 198)	(n = 397)	
Age (%)	Age ≤ 65	88 (44.22)	99 (50.00)	187 (47.10)	0.813
	Age >65	103 (51.76)	90 (45.45)	193 (48.61)	
	Unknown	8 (4.02)	9 (4.55)	17 (4.28)	
Sex (%)	Male	98 (49.25)	87 (43.94)	185 (46.60)	0.570
	Female	101 (50.75)	111 (56.06)	212 (53.40)	
Tumor stage (%)	I	105 (52.76)	112 (56.57)	217 (54.66)	0.493
	II	54 (27.14)	37 (18.69)	91 (22.92)	
	III	28 (14.07)	39 (19.70)	67 (16.88)	
	IV	11 (5.53)	9 (4.55)	20 (5.04)	
	Unknown	1 (0.50)	1 (0.51)	2 (0.50)	
T stage (%)	TX/T1	68 (34.17)	63 (31.82)	131 (33.00)	0.883
	T2/T3/T4	131 (65.83)	135 (68.18)	266 (67.00)	
M stage (%)	MX/M0	188 (94.47)	185 (93.43)	373 (93.95)	0.923
	M1	11 (5.53)	9 (4.55)	20 (5.04)	
	Unknown	0 (0.00)	4 (2.02)	4 (1.01)	
N stage (%)	NX/N0	128 (64.32)	136 (68.69)	264 (66.50)	0.609
	N1/N2/N3	71 (35.68)	61 (30.81)	132 (33.25)	
	Unknown	0 (0.00)	1 (0.51)	1 (0.25)	
OS status (%)	Living	131 (65.83)	131 (66.16)	262 (65.99)	0.998
	Deceased	68 (34.17)	67 (33.84)	135 (34.01)	

* *p*-value: chi-square test.

Subsequently, we explored the differences in mRNA expression between the GU and GS groups and found 42 differentially expressed genes (Figure 2D). Among these genes, *ROS1* was reported as an oncogenic driver in lung cancer (Rikova et al., 2007), and tyrosine kinase inhibitors (TKIs) targeting *ROS1* have been found to block tumor growth and provide clinical benefits for patients (D'Angelo et al., 2020; Shaw et al., 2014). *MAGEA2*, *MAGEA3*, *MAGEA4*, *MAGEA6*, and *MEGEA12*, members of the MAGE family, have been studied for their potential for cancer immunotherapy (Barker and Salehi, 2002). MAGEs were observed to be broadly expressed in many tumor types, such as lung cancer (Tajima et al., 2003; Gure et al., 2005; Kim et al., 2012), melanoma (Brasseur et al., 1995; Barrow et al., 2006), brain cancer (Scarcella et al., 1999), colorectal carcinoma (Mori et al., 1996), prostate cancer (Karpf et al., 2009), and breast cancer (Otte et al., 2001). To identify the potential functions of these 42 genes, we performed functional enrichment analysis, which indicated that these genes are involved mainly in the respiratory gaseous exchange by respiratory system (BP), histone deacetylase binding (MF) and endocytic vesicle, collagen trimer, lamellar body, multivesicular body, and clathrin-coated endocytic vesicle (CC) (Figure 2E).

### 3.2 Screening genome instability-derived genes as GULPsig

To explore the potential prognostic value of the 42 genome instability-related genes, we randomly divided all 397 samples into a training group (*n* = 199) and a testing group (*n* = 198) with the same size of living and deceased overall survival status in each group. Next, we conducted a univariate Cox regression analysis with samples in the training group to identify the prognostic-related genes (Supplementary Table S2). Among the genes, *IGF2BP3*, *SMC1B*, *IGF2BP1*, *CLDN6*, and *LY6K* were found to be closely associated with overall survival (Table 2, *p* < 0.05). The combined signature of the five genes was named GULPsig. *IGF2BP1* and *IGF2BP3* are both members of the IGF2BP family (Bell et al., 2013). *IGF2BP1* was found to promote proliferation (Rebucci et al., 2015), invasion (Zhou et al., 2015), and chemoresistance (Faye et al., 2015). In addition, its overexpression was associated with poor prognosis (Gu et al., 2004; Bell et al., 2015) in various types of cancer. There were also reports that the overexpression of *IGF2BP3* was closely related to poor prognosis in endometrial carcinoma (Fadare et al., 2013), oral squamous cell carcinoma (Lin et al., 2011), colorectal cancer (Shantha Kumara et al., 2015), ovarian cancer (Hsu et al., 2015), and lung adenocarcinoma (Guo et al., 2021). The overexpression of *SMC1B* was found to be associated with poor overall survival in hepatocellular carcinoma (Nie et al., 2021). *CLDN6* was also found to play a role in cancer cell migration and invasion in breast cancer (Song et al., 2019), hepatocellular carcinoma (Lu et al., 2021), and gastric cancer (Yu et al., 2019). *LY6K* was found to be a molecular marker for non-small-cell lung carcinoma (Ishikawa et al., 2007), head-and-neck squamous cell carcinoma (de Nooij-van Dalen et al., 2003), breast cancer (Kong et al., 2012), bladder cancer (Matsuda et al., 2011), and esophageal squamous cell carcinoma (Zhang et al., 2012).

**TABLE 2:** Univariate Cox regression analysis for five of the 42 genome instability-derived genes.

Genes	HR	95% CI for HR	*p*-value
IGF2BP3	1.23	1.10–1.38	<0.001
SMC1B	1.12	1.02–1.22	0.012
IGF2BP1	1.14	1.06–1.22	<0.001
CLDN6	1.10	1.03–1.18	0.006
LY6K	1.14	1.05–1.24	0.002

### 3.3 Construction of a risk model with GULPsig

**FIGURE 3:**
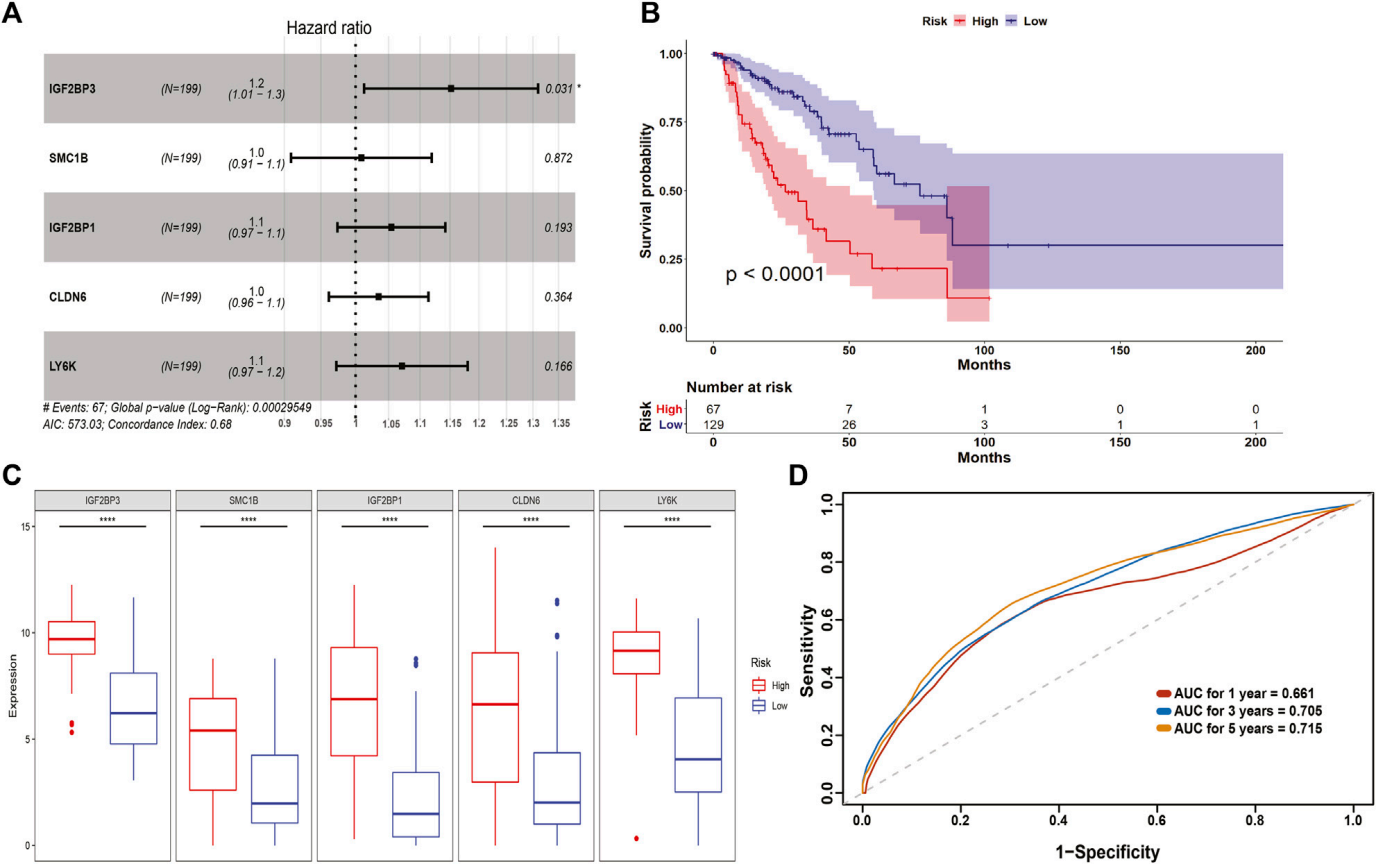
Identification of GULPsig for overall survival prediction. **(A)** Forest plot to show the hazard ratio for each gene in GULPsig. **(B)** Survival curve for the high- and low-risk groups. **(C)** Expression of each gene in GULPsig between the high- and low-risk groups. **(D)** 1-, 3-, and 5-year ROC curves for GULPsig.

We performed a multivariate Cox regression analysis (Figure 3A) and constructed a risk model based on the expression of GULPsig and the corresponding coefficients of multivariate Cox regression to evaluate the prognostic potential of GULPsig. The formula of the risk model is ORS = (0.141 × expression *IGF2BP3*) + (0.009 × expression *SMC1B*) + (0.053 × expression *IGF2BP1*) + (0.034 × expression *CLDN6*) + (0.069 × expression *LY6K*). The coefficients of GULPsig were all positive, suggesting that upregulated expressions of GULPsig are associated with poor prognosis. Then, we divided these patients into high- and low-risk groups according to the optimal cut-off threshold of 2.124. Patients whose scores were higher than the threshold were classified as high risk, and those whose scores were equal to or lower than the threshold were classified as low risk. We performed a chi-square test of association for the clinical characteristics between the high- and low-risk groups in the training group. A statistically significant association (*p* < 0.001) was found between the overall survival status and the risk groups, suggesting an association between the overall survival status and the risk groups (Supplementary Table S3).

To evaluate the performance of the prognostic risk model, we conducted the KM survival analysis to estimate the survival probability and the log-rank test to compare the survival curves of the high- and low-risk groups (Figure 3B). We found that the overall survival of the low-risk group was significantly better than that of the high-risk group (*p* < 0.001). We also checked the expression of each gene within GULPsig for both the high- and low-risk groups. The results showed that the expression of each gene in the high-risk group was significantly higher than that in the low-risk group (Figure 3C).

We assessed the specificity and sensitivity of GULPsig using ROC curve analysis and calculated AUC to evaluate its performance in overall survival prediction at 1, 3, and 5 years. The AUC for 1, 3, and 5 years was 0.661, 0.705, and 0.715, respectively, suggesting that GULPsig is better for predicting overall survival at 5 years (Figure 3D). In addition, the results of SVM and 10-fold cross-validation showed that GULPsig is effective in classifying patients into high- and low-risk groups (Supplementary Figure S3D).

### 3.4 Validation of GULPsig as a prognostic signature

**FIGURE 4:**
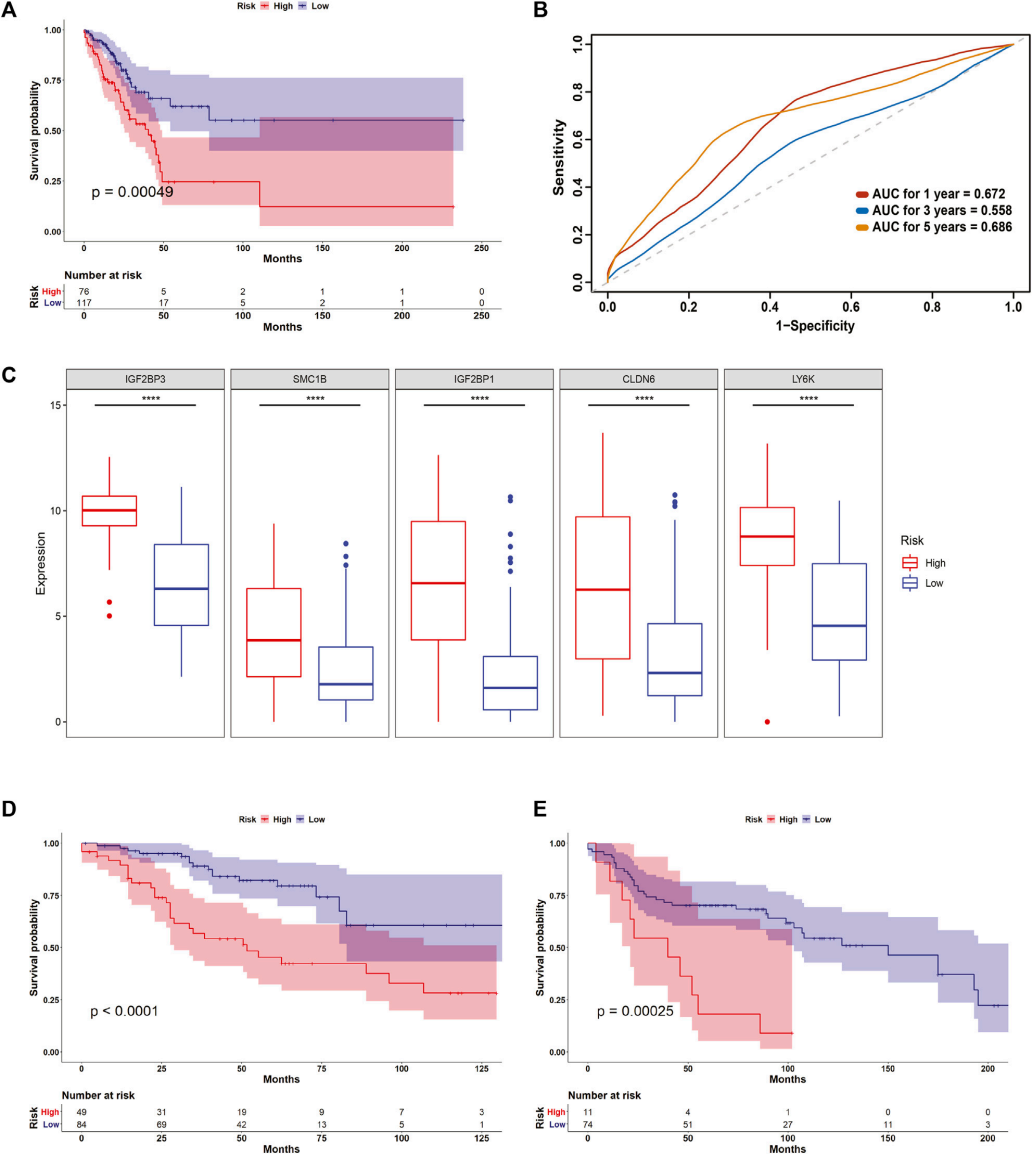
Validation of GULPsig as a prognostic signature. **(A)** Survival curve for the high- and low-risk groups in the testing dataset. **(B)** 1-, 3-, and 5-year ROC curves for GULPsig. **(C)** Expression of each gene in GULPsig between the high- and low-risk groups in the testing dataset. **(D)** Survival curve for the high- and low-risk groups in GSE42127. **(E)** Survival curve for the high- and low-risk groups in GSE30219.

To further evaluate the risk model with GULPsig, we divided the testing group into high- and low-risk groups according to the optimal cut-off threshold. In the testing group, 76 patients were classified into the high-risk group and 122 patients into the low-risk group. The chi-square test of association showed a significant association between the overall survival status and the risk groups (Supplementary Table S3, *p* < 0.001). Moreover, the results of the KM survival analysis and the log-rank test showed a significant difference between the high- and low-risk groups (Figure 4A, *p* < 0.01). The expression of each gene in GULPsig was significantly higher in the high-risk group than in the low-risk group (Figure 4C, *p* < 0.01). Moreover, the AUC for 1, 3, and 5 years in the testing group was 0.672, 0.558, and 0.686, respectively, suggesting that GULPsig was more effective in predicting overall survival at 5 years (Figure 4B). Additionally, the results of SVM and 10-fold cross-validation suggest the effectiveness of GULPsig in classifying patients into high- and low-risk groups in the testing dataset (Supplementary Figure S2B).

We also validated the prognostic value of the risk model with GULPsig on the TCGA dataset. Based on the optimal cut-off threshold, we classified 143 patients in the high-risk group and 254 patients in the low-risk group. We observed a significant association between the overall survival status and the risk groups (Supplementary Table S3, *p* < 0.001). The KM survival analysis and the log-rank test demonstrated that patients in the low-risk group had a significantly better prognosis than those in the high-risk group (Supplementary Figure S3A, *p* < 0.001). Furthermore, the expression of each gene in GULPsig was significantly higher in the high-risk group than in the low-risk group (Supplementary Figure S3B, *p* < 0.001). The AUC for 1, 3, and 5 years was 0.677, 0.634, and 0.697, respectively, indicating that GULPsig is better at predicting overall survival at 5 years (Supplementary Figure S3C). Moreover, SVM and 10-fold cross-validation showed the robustness of GULPsig for classifying patients into high- and low-risk groups (Supplementary Figure S3D). We used GSE42127 and GSE30219 to further validate the performance of GULPsig. When we applied the risk model to GSE42127, it classified 49 patients as high risk and 84 patients as low risk. The KM survival analysis and the log-rank test showed high significance between the high- and low-risk groups (Figure 4D, *p* < 0.001). For GSE30219, the model classified 11 patients as high risk and 74 as low risk. Similarly, a significant difference between the high-risk and low-risk groups was observed in the results of the KM analysis and the log-rank test (Figure 4E, *p* < 0.001).

### 3.5 Assessment of GULPsig as an independent prognostic signature

**FIGURE 5:**
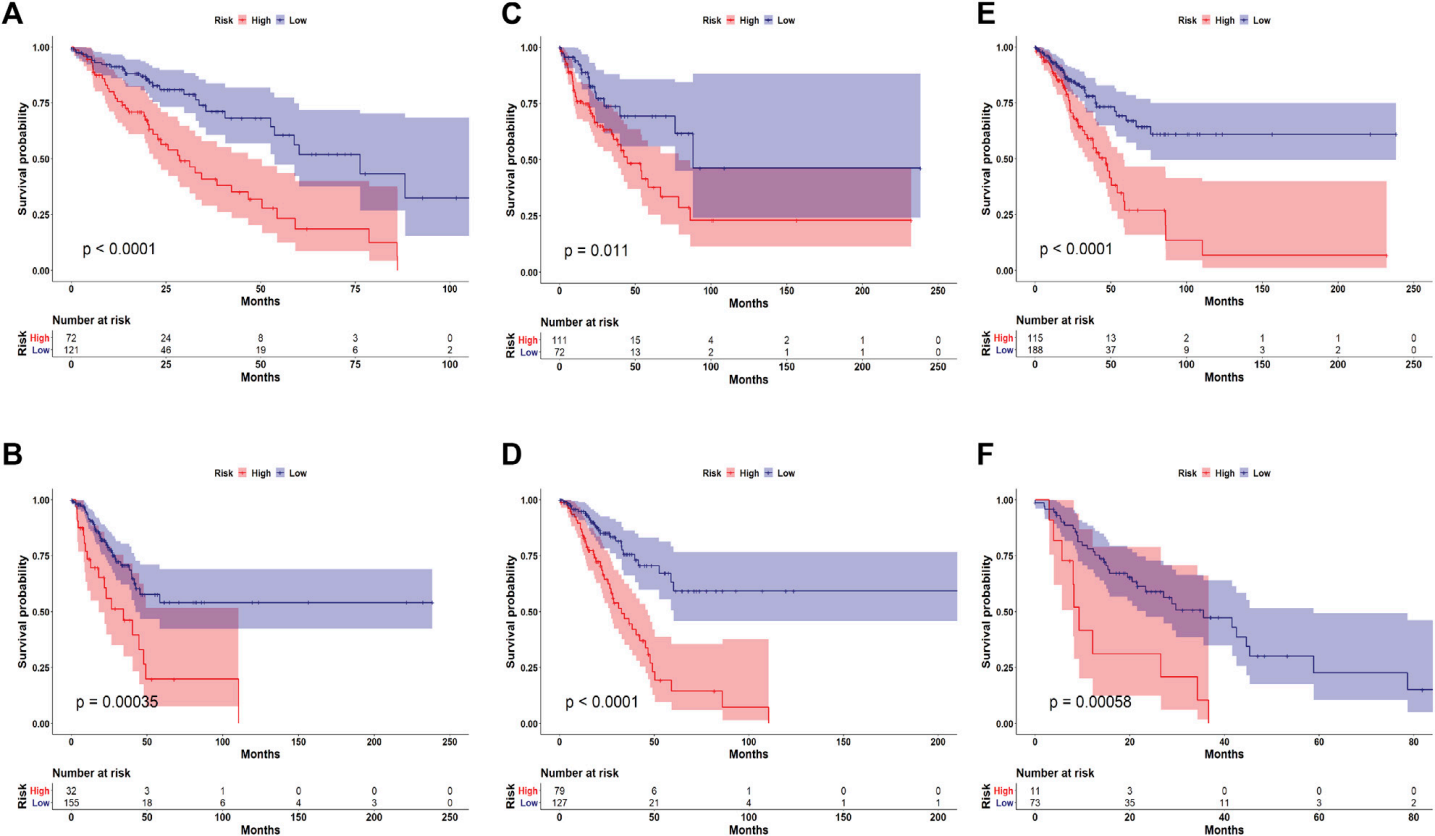
Assessment of GULPsig as an independent prognostic signature. **(A, B)** Survival curve for the high- and low-risk groups among patients aged >65 and ≤65. **(C, D)** Survival curve for the high- and low-risk groups among males and females. **(E, F)** Survival curve for the high- and low-risk groups among patients at tumor stage I/II and tumor stage III/IV.

To explore whether GULPsig was an independent prognostic signature from traditional clinical characteristics, we performed univariate and multivariate Cox regression analyses using the TCGA dataset. The results indicated that GULPsig (*p* < 0.001) is significantly associated with overall survival when adjustments are made for age, sex, tumor stage, T stage, M stage, and N stage (Table 3). Additionally, we divided the TCGA dataset into two groups based on age (>65 and ≤65), sex (male and female), and tumor stage (I/II and III/IV). Then, we used the risk model and categorized the patients in each group into high- and low-risk categories independently. The results demonstrated a significant difference between the high- and low-risk groups at age >65 (Figure 5A, *p* < 0.01), age ≤65 (Figure 5B, *p* < 0.01), male (Figure 5C, *p* < 0.05), female (Figure 5D, *p* < 0.01), tumor stage I/II (Figure 5E, *p* < 0.01), and tumor stage III/IV (Figure 5F, *p* < 0.01). These results demonstrate the independence of GULPsig as a prognostic signature, regardless of age, sex, and tumor stage.

**TABLE 3:** Univariate and multivariate Cox regression analyses.

Characteristics	Univariate analysis			Multivariate analysis		
	HR	95% CI	*p*-value	HR	95% CI	*p*-value
Age	1.01	0.99–1.02	0.470	1.02	1.00–1.04	0.024
Sex	1.04	0.74–1.47	0.812	0.96	0.67–1.36	0.807
Tumor stage	1.68	1.43–1.98	<0.001	1.51	1.16–1.97	0.002
T stage	1.62	1.30–2.03	<0.001	1.28	1.02–1.61	0.033
M stage	1.53	1.08–2.17	0.017	1.01	0.68–1.51	0.958
N stage	1.58	1.30–1.93	<0.001	1.01	0.76–1.33	0.964
GULPsig	2.08	1.58–2.74	<0.001	2.09	1.55–2.80	<0.001

### 3.6 Comparison of GULPsig with established prognostic signatures

**FIGURE 6:**
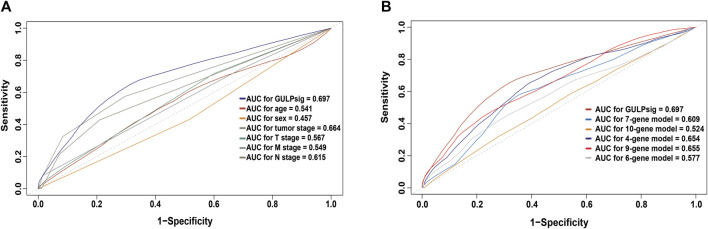
Comparison of GULPsig with established prognostic signatures. **(A)** ROC curves comparing GULPsig with traditional clinical characteristics. **(B)** ROC curves comparing GULPsig with the state-of-the-art prognostic signatures.

To further evaluate the performance of GULPsig, we compared GULPsig with traditional clinical characteristics, including age, sex, tumor stage, T stage, M stage, and N stage. By assessing the specificity and sensitivity using the ROC curve analysis and calculating the AUC, we found that the AUC of GULPsig (0.697) was larger than that of age (0.541), sex (0.457), tumor stage (0.664), T stage (0.567), M stage (0.549), and N stage (0.615), suggesting the better performance of GULPsig (Figure 6A). We also compared the performance of GULPsig with that of the state-of-the-art prognostic signatures, including the 7-gene signature (Al-Dherasi et al., 2021), 10-gene signature (Jiang et al., 2021), 4-gene signature (Liu et al., 2019), 9-gene signature (Zhang et al., 2019), and 6-gene signature (Xie and Xie, 2019). Still, the AUC of GULPsig (0.697) was larger than that of the 7-gene signature (0.609), 10-gene signature (0.524), 4-gene signature (0.654), 9-gene signature (0.655), and 6-gene signature (0.577), indicating that GULPsig performed better at overall survival prediction than the state-of-the-art prognostic signatures (Figure 6B).

## 4 Discussion

Traditional prognostic signatures for lung adenocarcinoma patients, such as age (Tas et al., 2013), sex (Radkiewicz et al., 2019), TNM stage, and tumor stage (Goldstraw et al., 2007), have provided valuable insights. However, the absence of molecular characteristics limits the comprehensiveness and accuracy of prognostic signatures. As our study has shown, incorporating genome instability-derived genes can substantially improve the accuracy of overall survival prediction for lung adenocarcinoma patients. We initially identified 42 genome instability-related genes by analyzing the gene expression profiles of patients with various genomic alternations. Using functional analysis, we found that these genes focused mainly on the biological process of the respiratory gaseous exchange by the respiratory system and the molecular function of histone deacetylase binding, which play vital roles in studies of molecular mechanisms for lung cancer (Valavanidis et al., 2013; Sanaei and Kavoosi, 2019; Rolfo et al., 2022; Seguin et al., 2022). Furthermore, we identified GULPsig, which includes *IGF2BP1*, *IGF2BP3*, *SMC1B*, *CLDN6*, and *LY6K*, from these 42 genome instability-related genes. Each gene in GULPsig has been reported to be involved in tumor development (Bell et al., 2013; Du et al., 2021; Nie et al., 2021; Guo D et al., 2022). We also constructed a risk model with GULPsig and stratified the patients into high- and low-risk groups based on the optimal cut-off threshold. Then, we validated the performance of GULPsig in various cohorts, including the testing group, TCGA dataset, and external datasets GSE42127 and GSE30219. These results further corroborated the effectiveness of GULPsig as a reliable and robust prognostic signature for lung adenocarcinoma patients. As we continued to evaluate the independence of GULPsig as a prognostic signature, irrespective of factors such as age, gender, and tumor stage, we embarked on a more comprehensive investigation of its potential clinical implications. By comparing it with established prognostic signatures, our findings revealed that GULPsig surpassed both traditional clinical characteristics and state-of-the-art prognostic signatures in performance, highlighting its potential for enhanced clinical applications in lung adenocarcinoma prognosis.

To the best of our knowledge, this study is the first to integrate somatic mutations, copy number variations, and fusion genes to comprehensively measure genome instability. Prior studies typically considered only one or two genomic alternations (Guo and Wang, 2021; Song et al., 2021; Chen et al., 2022). Our approach offers a more holistic representation of genome instability and its impact on prognostic signatures. Furthermore, while Guo C R et al. (2022) and Peng et al. (2021) identified genome instability-derived lncRNA signatures for lung adenocarcinoma patients, our study focused on mRNA-based signatures. An mRNA-based signature allows for a more direct assessment of gene expression, which, in turn, provides valuable insights into the functional consequences of genome instability. Additionally, focusing on mRNA can facilitate the identification of novel therapeutic targets and enrich our understanding of the molecular mechanisms driving tumor progression. This distinct approach underscores the unique contribution of our study to the field of lung adenocarcinoma prognostic research.

Our study has some limitations. The method of quantifying genome instability using genomic alterations needs further exploration. While we have pinpointed GULPsig as a potential prognostic signature for overall survival prediction, the hidden biological mechanisms related to GULPsig are still unclear, so further investigation is needed.

In conclusion, we identified GULPsig, consisting of *IGF2BP1*, *IGF2BP3*, *SMC1B*, *CLDN6*, and *LY6K*, as a potential prognostic signature for lung adenocarcinoma patients. However, further studies are required for clinical applications, such as the further validation of biological experiments and the exploration of hidden biological mechanisms.

## Data Availability

The original contributions presented in the study are included in the article/Supplementary Material; further inquiries can be directed to the corresponding author.
