# Supplementary material for: Genome instability-derived genes as a novel prognostic signature for lung adenocarcinoma
Source: Front Cell Dev Biol. 2023 Aug 16;11:1224069. doi: 10.3389/fcell.2023.1224069 (PMC10467266; doi:10.3389/fcell.2023.1224069)
Supplement: Supplementary file 1 [file DataSheet1.docx]

Supplementary Material

Genome Instability-Derived Genes as a Novel Prognostic Signature for Lung Adenocarcinoma

Xu Zhang^1^, Tak-Wah Lam^1^, Hing-Fung Ting^1*^

^1^Department of Computer Science, The University of Hong Kong, Hong Kong, China

*** Correspondence:** Hing-Fung Ting: hfting@cs.hku.hk

# Supplementary Figures


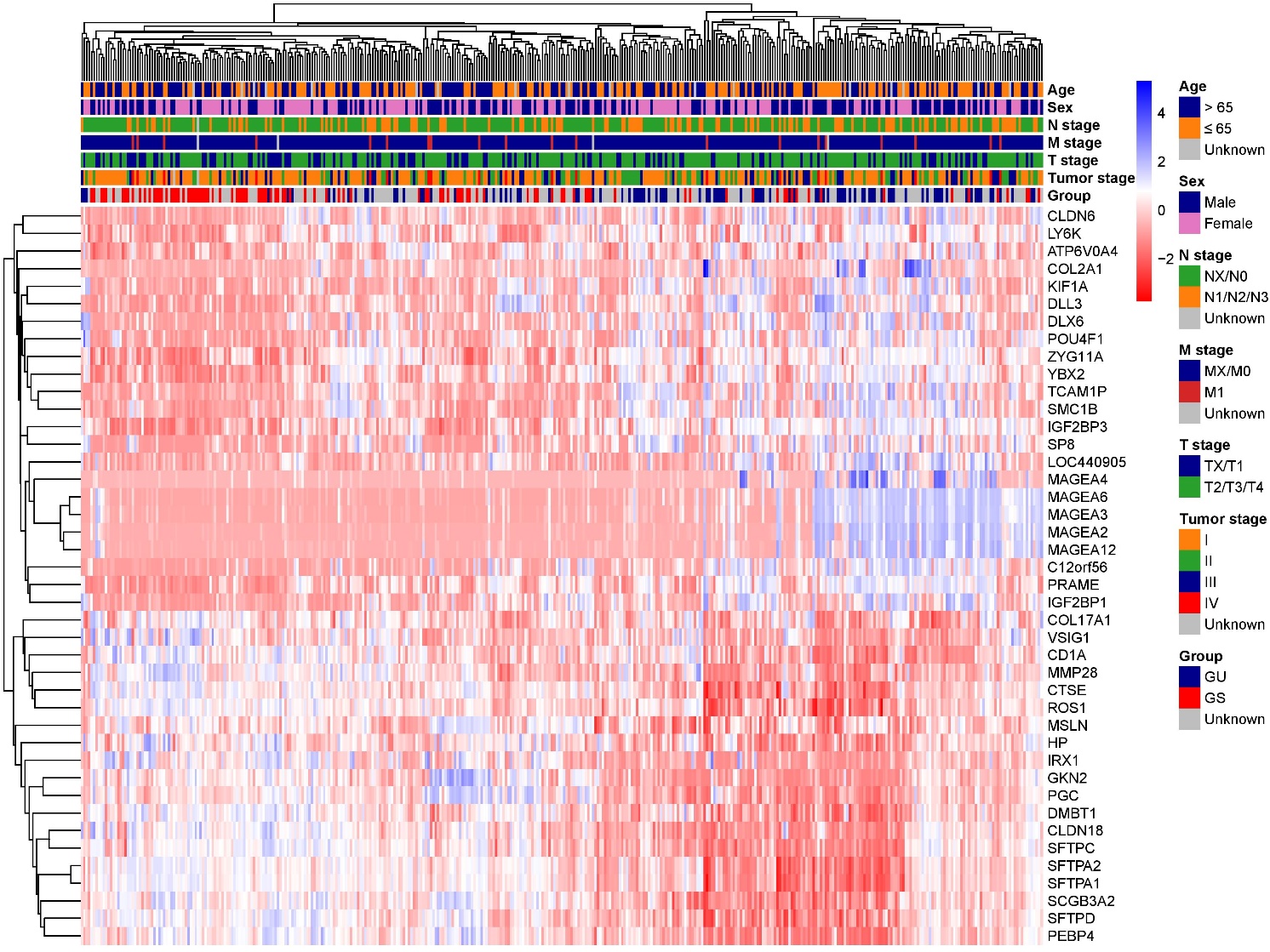


**Supplementary Figure 1 |** Hierarchical cluster of the 397 patients in the TCGA dataset with the expression of 42 genome instability-related genes.

**
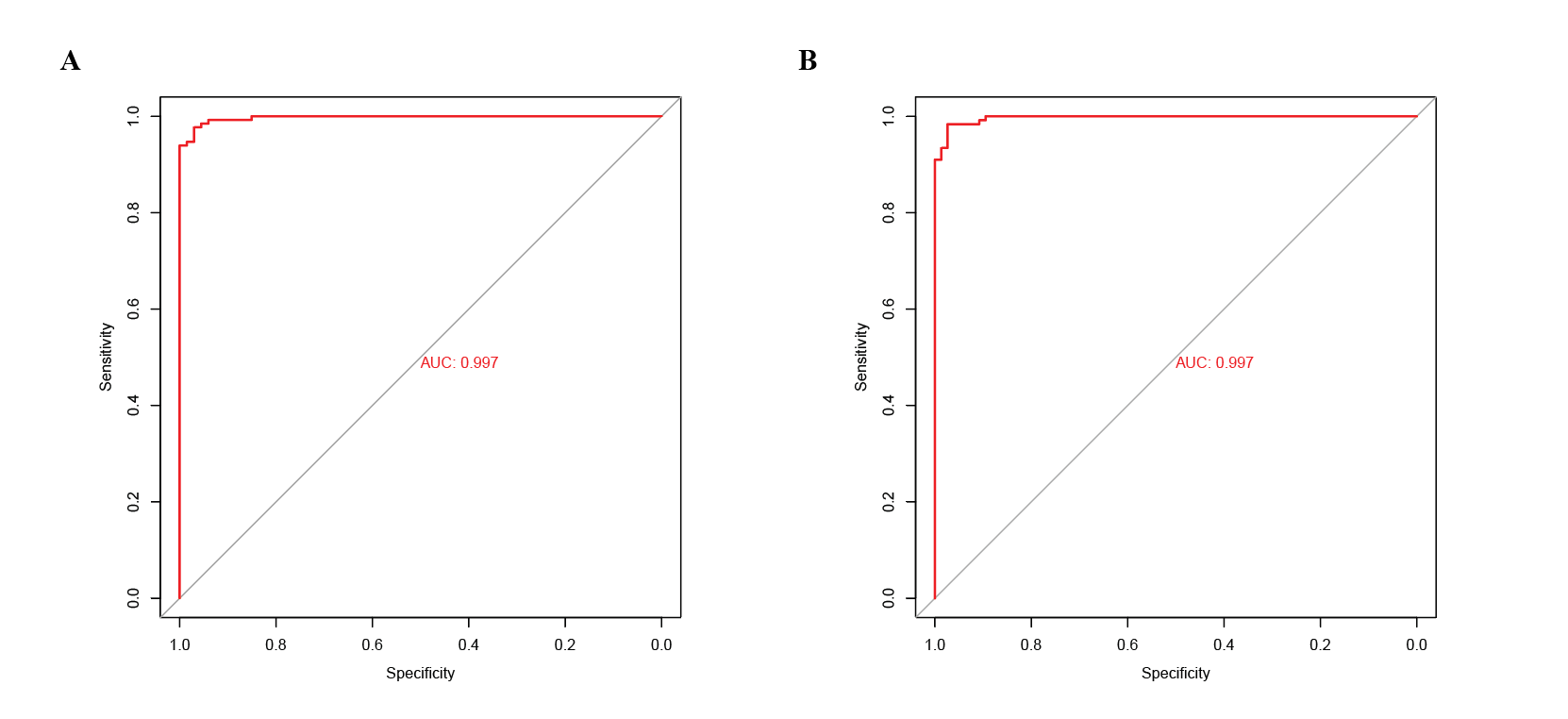
**

**Supplementary Figure 2 |** Evaluation of SVM classifiers. **(A)** ROC curve for the SVM classifier in the training group. **(B)** ROC curve for the SVM classifier in the testing group.


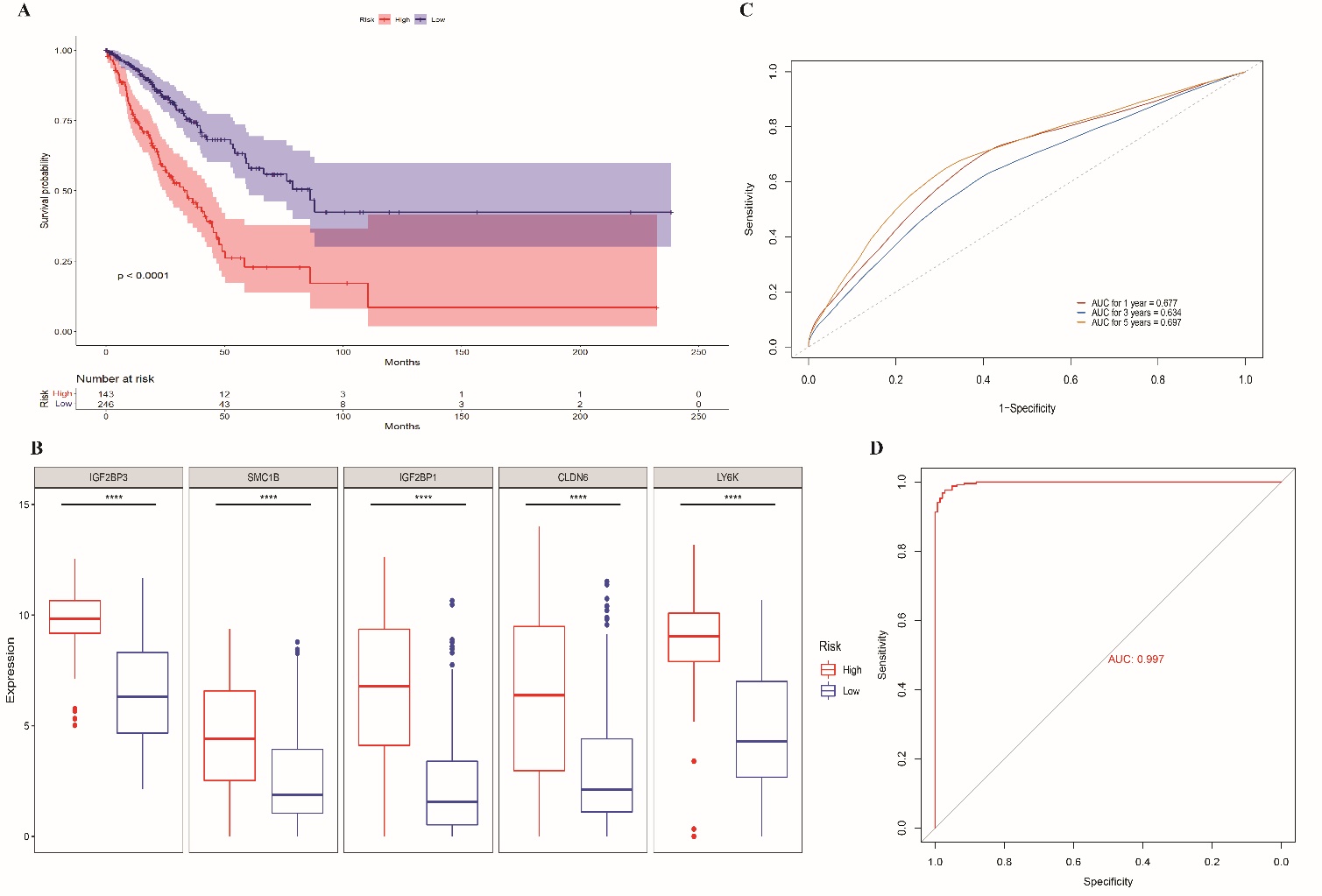
**Supplementary Figure 3 |** Validation of GULPsig as a prognostic signature in the TCGA dataset. **(A)** Survival curve for the high-risk and low-risk groups. **(B)** Expression of each gene in GULPsig between the high- and low-risk groups. **(C)** One-, three- and five-year ROC curves for GULPsig. **(D)** ROC curve for the SVM classifier.

# Supplementary Tables

**Supplementary Table S1 |** Clinical details for patients in the GU and GS groups in the training group

| **Characteristics** |  | **GU** | **GS** | **p value*** |
| --- | --- | --- | --- | --- |
|  |  | **(n = 99)** | **(n = 99)** |  |
| Age (%) | Age <= 65 | 54 (54.55) | 43 (43.43) | 0.149 |
|  | Age > 65 | 42 (42.42) | 53 (53.54) |  |
|  | Unknown | 3 (3.03) | 3 (3.03) |  |
| Sex (%) | Female | 55 (55.56) | 55 (55.56) | 1.000 |
|  | Male | 44 (44.44) | 44 (44.44) |  |
| Tumor stage (%) | I | 46 (46.46) | 59 (59.60) | 0.164 |
|  | II | 26 (26.26) | 20 (20.20) |  |
|  | III | 19 (19.19) | 15 (15.15) |  |
|  | IV | 8 (8.08) | 3 (3.03) |  |
|  | Unknown | 0 (0.00) | 2 (2.02) |  |
| T stage (%) | TX/T1 | 33 (33.33) | 36 (36.36) | 0.766 |
|  | T2/T3/T4 | 66 (66.67) | 63 (63.64) |  |
| M stage (%) | MX/M0 | 89 (89.90) | 94 (94.95) | 0.214 |
|  | M1 | 8 (8.08) | 3 (3.03) |  |
|  | Unknown | 2 (2.02) | 2 (2.02) |  |
| N stage (%) | NX/N0 | 62 (62.63) | 71 (71.72) | 0.187 |
|  | N1/N2/N3 | 37 (37.37) | 27 (27.27) |  |
|  | Unknown | 0 (0.00) | 1 (1.01) |  |
| OS stage (%) | Living | 67 (67.68) | 73 (73.74) | 0.435 |
|  | Deceased | 32 (32.32) | 26 (26.26) |  |

**p value: Chi square test*

**Supplementary Table S2 |** Univariate Cox regression analysis for the 42 genome instability-related genes

| **Genes** | **HR** | **95% CI for HR** | **p value** |
| --- | --- | --- | --- |
| CTSE | 0.98 | 0.91-1.06 | 0.622 |
| TCAM1P | 1.08 | 0.99-1.18 | 0.102 |
| POU4F1 | 1.04 | 0.94-1.15 | 0.439 |
| IGF2BP3 | 1.23 | 1.10-1.38 | <0.001 |
| YBX2 | 1.05 | 0.95-1.15 | 0.357 |
| SMC1B | 1.12 | 1.02-1.22 | 0.012 |
| ZYG11A | 1.06 | 0.95-1.19 | 0.292 |
| MMP28 | 1.02 | 0.92-1.14 | 0.642 |
| SP8 | 1.05 | 0.96-1.15 | 0.246 |
| ATP6V0A4 | 1.01 | 0.92-1.10 | 0.874 |
| ROS1 | 0.95 | 0.87-1.04 | 0.247 |
| C12orf56 | 1.05 | 0.97-1.15 | 0.233 |
| PRAME | 1.04 | 0.97-1.11 | 0.249 |
| COL2A1 | 0.99 | 0.90-1.08 | 0.761 |
| DLX6 | 1.07 | 0.97-1.18 | 0.197 |
| KIF1A | 1.00 | 0.94-1.06 | 0.984 |
| CD1A | 0.93 | 0.85-1.02 | 0.132 |
| LOC440905 | 1.08 | 0.98-1.18 | 0.113 |
| IRX1 | 0.99 | 0.90-1.09 | 0.834 |
| IGF2BP1 | 1.14 | 1.06-1.22 | <0.001 |
| DLL3 | 1.03 | 0.95-1.11 | 0.537 |
| SFTPD | 0.95 | 0.87-1.03 | 0.221 |
| PEBP4 | 0.97 | 0.90-1.05 | 0.462 |
| SFTPA2 | 0.97 | 0.90-1.03 | 0.317 |
| GKN2 | 0.99 | 0.92-1.07 | 0.854 |
| SCGB3A2 | 0.97 | 0.92-1.03 | 0.376 |
| CLDN18 | 0.97 | 0.90-1.04 | 0.372 |
| VSIG1 | 1.00 | 0.92-1.08 | 0.975 |
| DMBT1 | 0.97 | 0.90-1.03 | 0.306 |
| MAGEA6 | 1.03 | 0.98-1.09 | 0.217 |
| SFTPA1 | 0.96 | 0.90-1.02 | 0.191 |
| MAGEA3 | 1.03 | 0.98-1.09 | 0.242 |
| COL17A1 | 1.08 | 0.99-1.17 | 0.073 |
| HP | 0.96 | 0.89-1.04 | 0.356 |
| CLDN6 | 1.10 | 1.03-1.18 | 0.006 |
| LY6K | 1.14 | 1.05-1.24 | 0.002 |
| MAGEA2 | 1.02 | 0.96-1.08 | 0.601 |
| MAGEA12 | 1.01 | 0.95-1.08 | 0.714 |
| MAGEA4 | 1.06 | 1.00-1.13 | 0.060 |
| MSLN | 0.99 | 0.92-1.07 | 0.881 |
| SFTPC | 0.97 | 0.92-1.03 | 0.336 |
| PGC | 0.95 | 0.90-1.01 | 0.085 |

**Supplementary Table S3 |** Clinical details for patients classified into the high- and low-risk groups

|  |  | **Training group** | | | **Testing group** | | | **TCGA dataset** | | |
| --- | --- | --- | --- | --- | --- | --- | --- | --- | --- | --- |
|  |  | **(n = 199)** | | | **(n = 198)** | | | **(n = 397)** | | |
| **Characteristics** |  | **High risk** | **Low risk** | **p value** | **High risk** | **Low risk** | **p value** | **High risk** | **Low risk** | **p value** |
|  |  | **(n = 67)** | **(n = 132)** |  | **(n = 77)** | **(n = 121)** |  | **(n = 144)** | **(n = 253)** |  |
| Age (%) | age <= 65 | 33 (49.25) | 55 (41.67) | 0.523 | 42 (54.55) | 57 (47.11) | 0.616 | 75 (52.08) | 112 (44.27) | 0.327 |
|  | age > 65 | 33 (49.25) | 70 (53.03) |  | 34 (44.16) | 56 (46.28) |  | 67 (46.53) | 126 (49.80) |  |
|  | Unknown | 1 (1.49) | 7 (5.30) |  | 1 (1.30) | 8 (6.61) |  | 2 (1.39) | 15 (5.93) |  |
| Sex (%) | Female | 31 (46.27) | 70 (53.03) | 0.452 | 42 (54.55) | 69 (57.02) | 0.845 | 73 (50.69) | 139 (54.94) | 0.477 |
|  | Male | 36 (53.73) | 62 (46.97) |  | 35 (45.45) | 52 (42.98) |  | 71 (49.31) | 114 (45.06) |  |
| Tumor stage (%) | I | 27 (40.30) | 78 (59.09) | 0.065 | 40 (51.95) | 72 (59.50) | 0.001 | 67 (46.53) | 150 (59.29) | 0.075 |
|  | II | 25 (37.31) | 29 (21.97) |  | 14 (18.18) | 73 (60.33) |  | 39 (27.08) | 102 (40.32) |  |
|  | III | 11 (16.42) | 17 (12.88) |  | 19 (24.68) | 20 (16.53) |  | 30 (20.83) | 37 (14.62) |  |
|  | IV | 4 (5.97) | 7 (5.30) |  | 4 (5.19) | 5 (4.13) |  | 8 (5.56) | 12 (4.74) |  |
|  | Unknown | 0 (0.00) | 1 (0.76) |  | 0 (0.00) | 1 (0.83) |  | 0 (0.00) | 2 (0.79) |  |
| T stage (%) | TX/T1 | 15 (22.39) | 54 (40.91) | 0.015 | 20 (25.97) | 43 (35.54) | 0.211 | 35 (24.31) | 97 (38.34) | 0.006 |
|  | T2/T3/T4 | 52 (77.61) | 78 (59.09) |  | 57 (74.03) | 78 (64.46) |  | 109 (75.69) | 156 (61.66) |  |
| M stage (%) | MX/M0 | 63 (94.03) | 125 (94.70) | 1.000 | 71 (92.21) | 114 (94.21) | 0.989 | 134 (93.06) | 239 (94.47) | 0.896 |
|  | M1 | 4 (5.97) | 7 (5.30) |  | 4 (5.19) | 5 (4.13) |  | 8 (5.56) | 12 (4.74) |  |
|  | Unknown | 0 (0.00) | 0 (0.00) |  | 2 (2.60) | 2 (1.65) |  | 2 (1.39) | 2 (0.79) |  |
| N stage (%) | NX/N0 | 37 (55.22) | 91 (68.94) | 0.080 | 48 (62.34) | 88 (72.73) | 0.141 | 85 (59.03) | 179 (70.75) | 0.020 |
|  | N1/N2/N3 | 30 (44.78) | 41 (31.06) |  | 29 (37.66) | 32 (26.45) |  | 59 (40.97) | 73 (28.85) |  |
|  | Unknown | 0 (0.00) | 0 (0.00) |  | 0 (0.00) | 1 (0.83) |  | 0 (0.00) | 1 (0.40) |  |
| OS status (%) | Living | 31 (46.27) | 100 (75.76) | < 0.001 | 39 (50.65) | 92 (76.03) | < 0.001 | 70 (48.61) | 192 (75.89) | < 0.001 |
|  | Deceased | 36 (53.73) | 32 (24.24) |  | 38 (49.35) | 29 (23.97) |  | 74 (51.39) | 61 (24.11) |  |
